# Anomalous Lattice Dynamics in AgC_4_N_3_: Insights From Inelastic Neutron Scattering and Density Functional Calculations

**DOI:** 10.3389/fchem.2018.00544

**Published:** 2018-11-12

**Authors:** Baltej Singh, Mayanak K. Gupta, Ranjan Mittal, Mohamed Zbiri, Sarah A. Hodgson, Andrew L. Goodwin, Helmut Schober, Samrath L. Chaplot

**Affiliations:** ^1^Solid State Physics Division, Bhabha Atomic Research Centre, Mumbai, India; ^2^Homi Bhabha National Institute, Mumbai, India; ^3^Institut Laue-Langevin, Grenoble, France; ^4^Department of Chemistry, University of Oxford, Oxford, United Kingdom

**Keywords:** negative thermal expansion, linear compressibility, ab-initio, density functional theory, lattice dynamics, phonon, inelastic neutron scattering, 78.70.Nx, 63.20.-e, 65.40.-b

## Abstract

We have performed temperature dependent inelastic neutron scattering measurements to study the anharmonicity of phonon spectra of AgC_4_N_3_. The analysis and interpretation of the experimental spectra is done using *ab-initio* lattice dynamics calculations. The calculated phonon spectrum over the entire Brillouin zone is used to derive linear thermal expansion coefficients. The effect of van der Waals interaction on structure stability has been investigated using advanced density functional methods. The calculated isothermal equation of states implies a negative linear compressibility along the c-axis of the crystal, which also leads to a negative thermal expansion along this direction. The role of elastic properties inducing the observed anomalous lattice behavior is discussed.

## Introduction

In recent years, a few crystalline materials are found to exhibit expansion along certain crystallographic axes on application of static pressure (Goodwin et al., 2008a; Cairns et al., 2012, 2013; Cairns and Goodwin, 2015; Gupta et al., 2017; Yeung et al., 2017). This type of abnormal behavior is known as negative linear or area compressibility (NLC or NAC). The behavior is basically observed in materials with highly anisotropic bonding and open framework type structures (Goodwin et al., 2008a; Weng et al., 2008; Cairns et al., 2012, 2013; Cairns and Goodwin, 2015; Wang et al., 2016, 2017; Gupta et al., 2017; Zeng et al., 2017a,b; Yan et al., 2018). The anisotropic character of elastic properties is a measure of the magnitude of NLC (or NAC) in a material. The anisotropy in elastic properties of a material together with mode Grüneisen parameters give rise to the anisotropic thermal expansion (Gupta et al., 2016, 2017; Singh et al., 2017a; Mittal et al., 2018). Therefore, there is a close relation between the nature and magnitude of anisotropic thermal expansion and compressibility. Compounds which exhibit negative linear or area compressibilities are also found to show negative linear or area thermal expansion behavior along that particular directions (Conterio et al., 2008; Goodwin et al., 2008a,b, 2009; Gupta et al., 2017; Sapnik et al., 2018). However, the opposite is not true. Materials exhibiting the above properties are useful for pressure/temperature sensors, artificial muscles and can be used in piezoelectric applications (Burtch et al., 2017; Mirvakili and Hunter, 2017).

Many cyanide based metal organic flexible framework structures (Conterio et al., 2008; Goodwin et al., 2008a; David et al., 2010; Mittal et al., 2012; Cairns et al., 2013; Gupta et al., 2017; Ovens and Leznoff, 2017; Sapnik et al., 2018) like ZnAu_2_(CN)_4_, M_3_Co(CN)_6_ and MAuX_2_(CN)_2_ where M = H, Au, Ag, Cu, Fe and X = CN, Cl, Br etc., show large NLC and NTE behaviors. These stem from their special structure and bonding. In case of ZnAu_2_(CN)_4_, the NLC, and NTE along hexagonal c-axis arise from the anharmonic nature of the low energy optic phonon modes involving bending of the –Zn–NC–Au–CN–Zn– linkage, mimicking the effect of a spring in terms of compression upon heating and elongation (Gupta et al., 2017; Wang et al., 2017) under a hydrostatic pressure. The mechanism of the deformation of a wine rack structure produces NLC and NTE behavior in many compounds (Goodwin et al., 2008a; David et al., 2010; Cairns et al., 2012; Zeng et al., 2017a; Sapnik et al., 2018). Negative area compressibility and negative area thermal expansion are found to arise from the sliding of atomic layers as a function of pressure or temperature (Zeng et al., 2017b).

Experimental tools like x-ray diffraction and neutron diffraction are generally used to obtain temperature and pressure dependent lattice parameters (Goodwin et al., 2008a,b, 2009; Cairns et al., 2012, 2013; Hodgson et al., 2014; Cairns and Goodwin, 2015). However, the mechanism at the origin of this type of abnormal behavior can only be understood by studying the microscopic dynamics at the atomic level. *Ab-initio* density functional theory provides the pressure dependent phase diagram of these compounds and can be used to obtain the movements of atoms at different pressures, giving rise to negative linear or area compressibilities (Gupta et al., 2013, 2016, 2017; Singh et al., 2017a,b; Mittal et al., 2018). On the other hand, phonons in the entire Brillouin zone calculated using the *ab-initio* lattice dynamics in combination with the elastic properties have successfully been used to reproduce the experimental values of anisotropic linear thermal expansion coefficients (Gupta et al., 2013, 2016, 2017; Singh et al., 2017a,b; Mittal et al., 2018). This methodology enables us to identify specific phonon modes responsible for the negative thermal expansion behavior. Moreover, *ab-initio* techniques, being highly accurate as compared to empirical potentials based techniques, have now been used to identify and model new materials exhibiting anomalous lattice behavior (Lazar et al., 2015; Singh et al., 2018).

Recently, a combination of experimental phonon density of states coupled with *ab-initio* calculations are used for understanding the anomalous lattice response in a few metal organic framework compounds (Duyker et al., 2013; Hermet et al., 2013; Kamali et al., 2013; Gupta et al., 2016, 2017). These calculations are found to provide fair agreement of calculated and experimental phonon spectra. The structure of metal organic framework compounds has highly anisotropic bonding. The van der Waals interactions are found to play a very important role in stabilizing the structure and dynamics of these compounds (Conterio et al., 2008; Kamali et al., 2013; Gupta et al., 2017). However, the calculated temperature dependence of lattice parameters is not in satisfactory agreement with the measurements. The unavailability of accurate van der Waals dispersion interactions in the density functional theory may be responsible for this disagreement. However, the calculations are useful to qualitatively understand the mechanism responsible for anomalous lattice response of these compounds which are directly related to the low energy phonon modes in these compounds.

**Figure 1:**
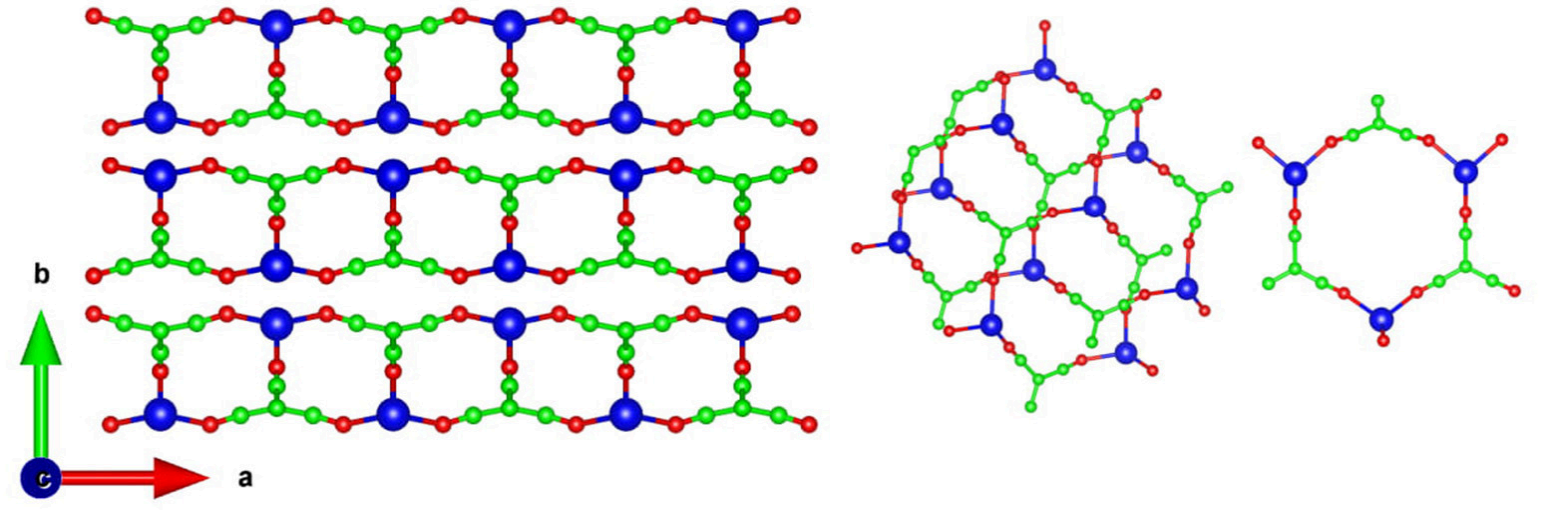
**(Left)** The crystal structure of AgC_4_N_3_ projected in the ab plane. **(Right)** The building block consisting of 18 interpenetrating membered rings, and forming the ab plane of the structure. Key: C-Green, N-Red, and Ag-Blue.

Silver (I) tricyanomethanide (Figure 1), AgC_4_N_3_, exhibits negative area compressibility and negative area thermal expansion in the a-c plane (Hodgson et al., 2014). The experimental temperature dependent lattice parameters were obtained from the single crystal X-ray diffraction experiments (Hodgson et al., 2014) while the pressure dependence of lattice parameter were obtained from the powder neutron diffraction experiments (Hodgson et al., 2014). The experimental X-ray and neutron diffraction techniques show large values of linear thermal expansion coefficients, α_a_ = −48 × 10^−6^ K^−1^, α_b_ = 200 × 10^−6^ K${}_{,}^{-1}$ α_c_ = −54 × 10^−6^ K^−1^. We have used the *ab-initio* calculated equation of states to obtain the pressure dependence of the lattice parameters. The anisotropic pressure dependence of phonon spectra over the entire Brillouin zone is used to extract the anisotropic Grüneisen parameters within the quasiharmonic approximation framework. The temperature dependent phonon spectra are obtained from inelastic neutron scattering (INS) measurements. The calculated Grüneisen parameters and elastic constants are used to estimate linear thermal expansion coefficients. We find that the calculations reproduce the NLC and NTE behavior along the c-axis; however, the same along the a-axis are not reproduced. We have performed the analysis of specific phonon modes to gain insights into the atomic level mechanisms at the origin of the observed negative thermal expansion behavior along the c-axis.

## Experimental and computational details

INS measurements on about 2 cc of polycrystalline sample of AgC_4_N_3_ were carried out on the direct-geometry cold-neutron time-of-flight time-focusing spectrometer IN6 at the Institut Laue Langevin (ILL, Grenoble, France). The spectrometer is equipped with a large detector bank covering a wide range of about 13–114° of scattering angle. Data were collected at 150, 225, 300, and 400 K, in the neutron energy gain setup and high-resolution mode, using an incident wavelength of 5.12 Å (3.12 meV). In the incoherent one-phonon approximation (Carpenter and Price, 1985; Price and Skold, 1986), the measured scattering function *S*(*Q*,*E*), as observed in the neutron experiments, is related to the phonon density of states g^(n)^^(^E) as follows:

(1) $${\text{g}}^{\left(\text{n}\right)}(E)=\text{A}<\frac{{\text{e}}^{\text{2W}(\text{Q})}}{{\text{Q}}^{2}}\frac{\text{E}}{\text{n}(\text{E},\text{T})\text{+}\frac{1}{2}\pm \frac{1}{2}}\text{S}(\text{Q},\text{E})>$$

(2) $$\begin{array}{rcl} {\text{g}}^{\text{n}}\left(E\right) & = & \text{B}\underset{k}{\sum }\left\{\frac{4\pi {\text{b}}_{\text{k}}^{2}}{{\text{m}}_{\text{k}}}\right\}{\text{g}}_{{}^{\text{k}}}\left(E\right) \end{array}$$

where the + or − signs correspond to energy loss or gain of the neutrons, respectively and $\text{n}\left(E,T\right)={\left[\exp\left(E\text{/}{\text{k}}_{B}\text{T}\right)-1\right]}^{-1}$. *A* and *B* are normalization constants. *b*_*k*_, *m*_*k*_, and *g*_*k*_(*E*) are, respectively, the neutron scattering length, mass, and partial density of states of the *k*th atom in the unit cell. The quantity between < > represents suitable average over all *Q* values at a given energy. 2*W*(*Q*) is the Debye-Waller factor averaged over all the atoms. The weighting factors $\frac{4\pi b_{k}^{2}}{m_{k}}$ in the units of barns/amu for C, N, and Ag are: 0.4625, 0.8221, and 0.0462, respectively. The values of neutron scattering lengths for various atoms can be found from Sears (1992).

The Vienna based *ab-initio* simulation package (VASP) (Kresse and Furthmüller, 1996; Kresse and Joubert, 1999) is used to carry out the total energy calculation based on plane-wave pseudo potential methods. The calculations are performed using projected augmented wave (PAW) formalism of Kohn- Sham density functional theory with generalized gradient approximation (GGA) for exchange correlation as given by Perdew, Becke and Ernzerhof (Perdew et al., 1996, 1997). K-point sampling was performed using 4 × 4 × 4 mesh Monkhorst-pack scheme (Monkhorst and Pack, 1976) with a plane wave energy cutoff of 900 eV. Different schemes allowing incorporating the effect of van der Walls (vdW) interaction are available in VASP, using different approximations. The phonon frequencies in the entire Brillouin zone are estimated using finite displacement method, within the direct method approach, as implemented in PHONON5.2 (Parlinksi, 2003). Hellman-Feynman forces are calculated by the finite displacement of 0.03 Å.

Thermal expansion calculation is done using pressure dependence of phonon frequencies in the entire Brillouin zone within the quasi-harmonic approximation. Each phonon mode of energy *E*_*qj*_ (*jth* phonon mode at point q in the Brillouin zone) contributes to the thermal expansion coefficient, which is given by the following relation for an orthorhombic system (Grimvall, 1999):

$$\begin{array}{l} \alpha_{l}(T)=\frac{1}{V_{0}}\underset{q,j}{\sum^{\text{​}}}C_{l^{\prime }}(q,j,T)[s_{l1}\Gamma_{a}+s_{l2}\Gamma_{b}+s_{l3}\Gamma_{c}]\;,\;\\ \;l,\;l^{\prime }=a,b,\;c\&l\neq l^{\prime } \end{array}$$

Where s_ij_ are elements of elastic compliances matrix, *s* = C^−1^ at constant temperature *T* = 0 K, *V*_0_ is volume at 0 K and C${}_{l^{\prime }}$(q,*j*,*T*) is the specific heat at constant strain due to *jth* phonon mode at point ***q*** in the Brillouin zone. The mode Grüneisen parameter of phonon energy *E*_*q, j*_ is given as Grüneisen and Goens (1924),

$$\begin{array}{l} \Gamma_{l}\left(E_{q,j}\right)=-{\left(\frac{\partial lnE_{q,j}}{\partial lnl}\right)}_{l^{\prime }};\;l,\;l^{\prime }=a,b,\;c\&l\neq l^{\prime } \end{array}$$

The volume thermal expansion coefficient for an orthorhombic system is given by:

$$\begin{array}{l} \alpha_{V}=\left(\alpha_{a}+\alpha_{b}+\alpha_{c}\right) \end{array}$$

## Results and discussion

The structure of AgC_4_N_3_ is a layered-like network topology along the b-axis (Hodgson et al., 2014). Ag^+^ cation is coordinated to three N atoms to form trigonal arrangements. In the a–c plane it forms a crossing network made up of 18-member (Ag_3_C_9_N_6_) rings (Figure 1). The layers in the a–c planes interact along the b-axis through a weak vdW interaction, acting between and N atoms. We have performed the relaxation of the crystal structure with and without including vdW interactions. These calculations without vdW interactions overestimated the b lattice parameter by 22% in comparison to the experimental value (Table 1). As shown in Table 1, we have initially optimized the structure including various available van der Waals schemes. We found that including vdW interaction describes better the structure whose calculated lattice parameters agree with the experimental (Cairns et al., 2013) values (Table 1). The vdW interaction has been considered using the optB88-vdW functional scheme of the vdw-DFT method (Dion et al., 2004; Jirí et al., 2010; Klimeš et al., 2011). The calculations produce the calculated volume within 1% of the experimental (Hodgson et al., 2014) value. The ambient condition structure is well-reproduced by the calculations.

**Table 1:** The comparison of experimental (Single crystal X-ray diffraction Hodgson et al., 2014 at 100 K and ambient pressure) and calculated structural parameters, using various pseudopotentail corrections for the dispersion interactions.

**Lattice parameters/Optimization scheme**	**Expt. (100 K)**	**GGA**	**GGA + optB88**	**GGA + optB86b**	**GGA + optPBE**	**GGA + revPBE**	**GGA + DFT-D2, Grimme**
a (Å)	8.0636	8.1317	7.9007	7.8991	7.8961	7.9435	8.421
b(Å)	9.7766	12.0189	9.6641	9.6466	10.0359	10.5742	9.418
c(Å)	6.2791	6.0829	6.4314	6.4110	6.4808	6.5248	6.020
V(Å^3^)	495.01	594.5	491.1	488.5	513.6	548.1	477.5
**(x, y, z)**							
C1	0.25	0.25	0.25	0.25	0.25	0.25	0.25
	0.4525	0.4622	0.4449	0.4456	0.4454	0.4463	0.4577
	0.9888	0.9113	0.9100	0.9103	0.9118	0.9133	0.9088
C2	0.5973	0.6005	0.5960	0.5958	0.5956	0.5963	0.6048
	0.6570	0.6368	0.6580	0.6582	0.6570	0.6545	0.6530
	0.2735	0.1962	0.1964	0.1970	0.1944	0.1921	0.2081
C3	0.75	0.75	0.75	0.75	0.75	0.75	0.75
	0.6219	0.6058	0.6251	0.6247	0.6240	0.6220	0.6171
	0.1798	0.0987	0.1007	0.1017	0.1001	0.0987	0.1100
N1	0.25	0.25	0.25	0.25	0.25	0.25	0.25
	0.5139	0.5210	0.5020	0.5027	0.5019	0.5024	0.5207
	0.8313	0.7578	0.7499	0.7493	0.7539	0.7586	0.7413
N2	0.4718	0.4751	0.4660	0.4651	0.4654	0.4674	0.4806
	0.6835	0.6622	0.6857	0.6868	0.6853	0.6824	0.6819
	0.3470	0.2771	0.2732	0.2725	0.2699	0.2676	0.2827
Ag	0.25	0.25	0.25	0.25	0.25	0.25	0.25
	0.64736	0.6244	0.6301	0.6314	0.6303	0.6295	0.6544
	0.5546	0.4678	0.4819	0.4816	0.4876	0.4919	0.4402

*Various van der Waals dispersion interaction schemes (Dion et al., 2004; Jirí et al., 2010; Klimeš et al., 2011) are used in the structure optimization*.

### Phonon spectra

**Figure 2:**
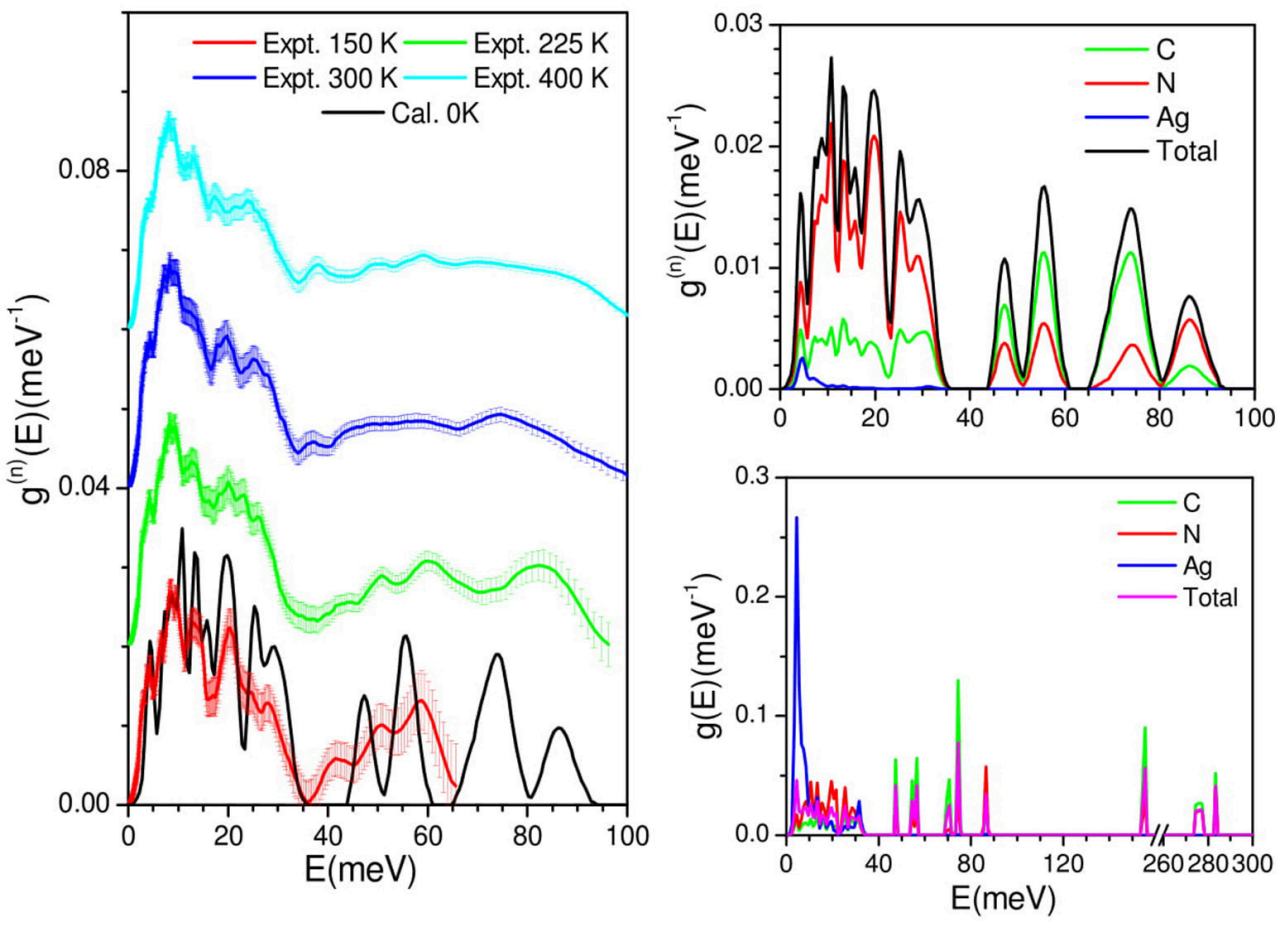
**(Left)** Comparison of DFT calculation, and experimental phonon density of states of AgC_4_N_3_. For clarity, the experimental spectra are shift vertically. **(Right: Top)** The calculated neutron-weighted total density of states and the partial contributions from various atoms of AgC_4_N_3_. The calculated neutron spectra have been broadened corresponding to the experimental resolution. **(Right: Bottom)** The calculated partial density of states from various atoms of AgC_4_N_3_, showing the full energy range to account for the C-N modes bending and stretch.

The measured phonon spectra at 150, 225, 300, and 400 K are shown in Figure 2. The INS measurements are performed in the neutorn energy gain mode. At 150 K, due to the population factorthe maximum energy transfer islimited to 65 meV. As temperature increases, modes at higher frequencies are populated, extending the energy range upto 100 meV. All the peaks in the phonon spectra show broadening with increase in temperature. However, peaks above 40 meV are found to show large temperature dependence. The broadening in the spectra reflects the explicit anharmonicity of phonons, which is expected with increase of temperature due to the increase of phonon-phonon interactions.

We have calculated the phonon spectra of this compound over the entire Brillouin zone. The calculated (0 K) phonon density of state is compared with the experimentally measured inelastic neutron scattering spectra at 150 K. The calculated neutron weighted phonon density of statesa grees very well with the experimental data (Figure 2). The level of agreement between the calculated and experimental phonon density of states of AgC_4_N_3_ is similar to that in many previous studies on metal cyanides framework compounds (Gupta et al., 2016, 2017), which is quite good considering the use of incoherent approximation (Carpenter and Price, 1985; Price and Skold, 1986) in the calculation of neutron weighted phonon spectrum. Therefore, the calculations can be further used for the microscopic understanding and interpretation of the peaks in the phonon spectra. The calculated partial phonon density of states show that Ag atoms contribute only tothe low energy part of the spectra, while C and N atomic vibrations contribute to the entire energy range. The low energy peak in the spectra around 5 meV arises from the vibrational motion of all the atomic species. The vibrations below 35 meV are dominated by the contribution from N atoms, while above 45 meV, the contributions upto 80 meV are mainly duetoC atoms. The calculated spectra in the 40–60 meV energy range shows a deviation from the observation. There could be two reasons for this deviation: (i) small mismatch between the calculated and experimental bond lengths due to limitation of density functional used for exchange correlations and dispersion interactions and (ii) this region is very sensitive to temperature so the mismatch may be due to the comparison of 0 K calculations with the meaurements performed at 150 K. As the vibrations of C≡N bond occur with very high energy of order 270 meV, they do not undergo any significant changes, but they are well-captured by our calculations. The peak in the calculated spectrumaround 165 meV corresponds to the bending vibrations of –C≡N units.

**Figure 3:**
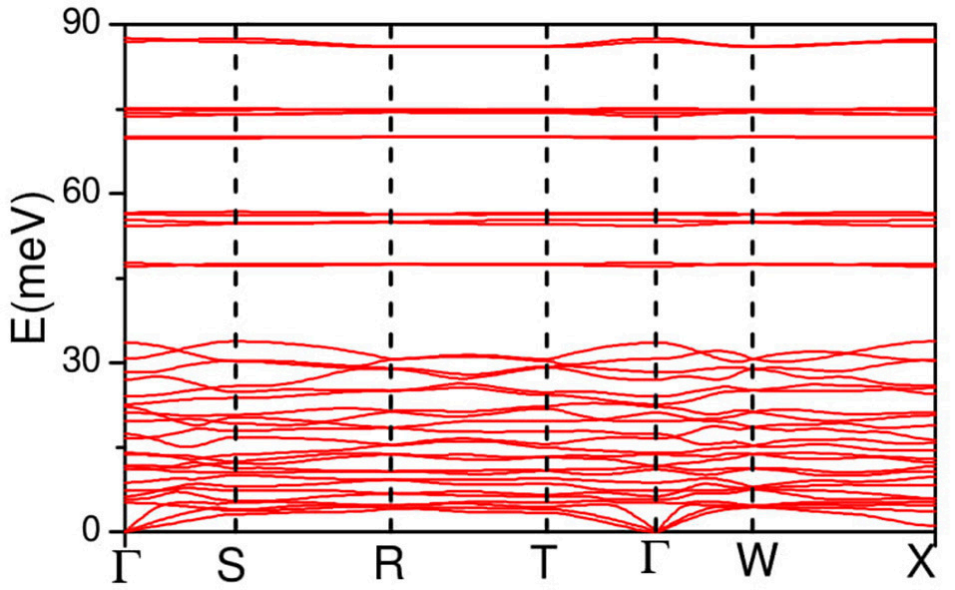
The calculated phonon dispersion curves along the high symmetry directions in the Brillouin zone AgC_4_N_3_. The Bradley-Cracknell notation is used for the high-symmetry points; Γ(0, 0, 0), S(1/2, 0, 0), R(0, 1/2, 0), T(1/2 0 0), W(1/4, 1/4, 1/4), and X(1/2, −1/2, 1/2).

The calculated phonon dispersion curves along the high symmetry directions in the Brillouin zone are shown in Figure 3. The phonon dispersionss are almost flat around 48, 55, 70, and 88 meV, which give rise to intense peaks in the calculated phonon density of states (Figure 2). This implies a very strong short range interactions among the atoms contributing to these vibrational modes.

### Anisotropic compressibility behavior

**Figure 4:**
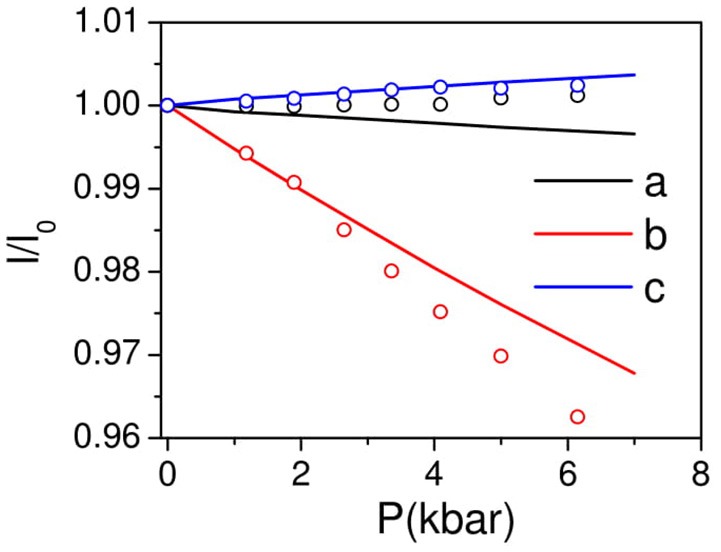
The calculated (solid lines) pressure dependence of unit cell lattice parameters of AgC_4_N_3_ compared with the experimental data (circles). The experimental data is available from 1 to 6 kbar. The data was extrapolated to 0 kbar and used for estimation of *l/l*_0_.

The pressure dependent equation of state of AgC_4_N_3_ has been calculated (Figure 4) by applying a uniform hydrostatic pressure to look at the resulting change in the lattice parameters. The *a* and *b* lattice parameters are found to show a usual, but anisotropic positive linear compressibility, while the c lattice parameter shows a negative linear compressibility. Further the elastic constants are calculated using the symmetry-general least square method (Le Page and Saxe, 2002) as implemented in VASP-5.4. The values are derived from the strain–stress relationships obtained from finite distortions of the equilibrium lattice. For small deformations the elastic domain of the solid is conserved and a quadratic dependence of the total energy with respect to the strain is expected (Hooke's law). The number of components (Mouhat and Coudert, 2014) of the elastic constant tensor is related to the symmetry of the crystal symmetry. The calculated elastic constant, *C*_ij_ (in kbar) and compliance, *s*_ij_ (10^−4^ kbar^−1^) matrices are:

$$\begin{array}{lll} C_{ij} & = & \left(\begin{array}{cccccc} 588 & 186 & 508 & 0 & 0 & 0\\ 186 & 195 & 214 & 0 & 0 & 0\\ 508 & 214 & 645 & 0 & 0 & 0\\ 0 & 0 & 0 & 100 & 0 & 0\\ 0 & 0 & 0 & 0 & 20 & 0\\ 0 & 0 & 0 & 0 & 0 & 42 \end{array}\right),\\ s_{ij} & = & \left(\begin{array}{cccccc} 54.0 & -7.3 & -40.1 & 0 & 0 & 0\\ -7.3 & 81.7 & -21.4 & 0 & 0 & 0\\ -40.1 & -21.4 & 54.2 & 0 & 0 & 0\\ 0 & 0 & 0 & 99.9 & 0 & 0\\ 0 & 0 & 0 & 0 & 502.4 & 0\\ 0 & 0 & 0 & 0 & 0 & 238.9 \end{array}\right) \end{array}$$

It can be seen that the value of longitudinal elastic constants C_11_ and C_33_ are about three times in comparison to that of C_22_, indicating that b-axis is highly compressible in comparison to a- and c- axis. Qualitatively, this agrees quite well with the experimental (Hodgson et al., 2014) pressure dependence of unit cell lattice parameters of AgC_4_N_3_ which show that the response to the pressure along b-axis is very large in comparison to that along a- and c-axes. It should be noted that the a–c plane of the crystal consists of layers containing 18-membered (Ag_3_C_9_N_6_) interpenetrating rings. The layers in the a–c plane are stacked along b-axis which indicates that the compound may have highly anisotropic elastic response. The calculations confirm that at ambient pressure there is large anisotropy in bonding along various crystal axes which gives rise to highly anisotropic elastic properties in this compound.

Further in order to understand the compressibility behavior along the orthorhombic axes of AgC_4_N_3_ we have calculated

$$\begin{array}{l} \begin{array}{c} X_{a}=S_{11}+S_{12}+S_{13},X_{b}=S_{12}+S_{22}+S_{23}\;\&\\ X_{c}=S_{13}+S_{23}+S_{33} \end{array} \end{array}$$

Where *X*_*i*_ (*i* = *a, b, c*) are the compressibilities of crystal along various crystallographic axes. For negative compressibility, the values of *X*_*a*_*, X*_*b*_*, and X*_*c*_ should be negative along the respective axis (Weng et al., 2008). We found that *X*_*a*_ = *6.4* × 10^−4^ kbar^−1^, *X*_*b*_ = *53.0* × 10^−4^ kbar^−1^*, X*_*c*_ = −7.3 × 10^−4^ kbar^−1^. These calculations imply negative linear compressibility along c-axis. However, the experimentally observed compressibility behavior along the *a*-axis is not obtained (Figure 4) from the calculations. This could be due to limitation of vdW DFT to account properly for the pressure dependent dispersion interactions in this particular structure geometry. It should be noted that a similar vdW density functional has been successfully used to obtain the experimentally observed thermal expansion behavior in many cyanide framework compounds (Kamali et al., 2013; Gupta et al., 2017).

**Figure 5:**
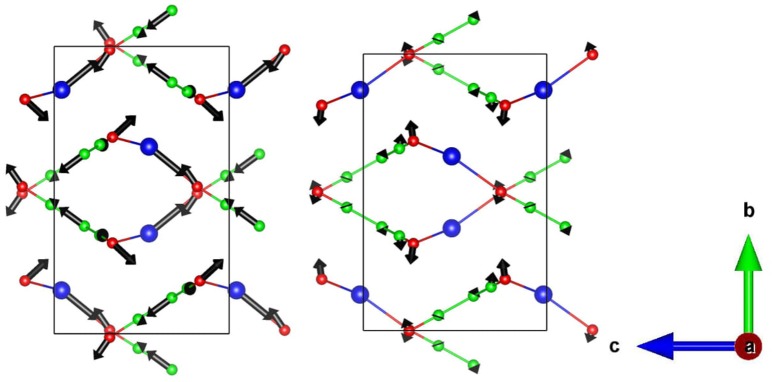
The displacement vectors corresponding to the difference (× 35) in atomic coordinates of **(Left)** experimental structures at 1.18 and 6.15 kbar, and **(Right)** calculated structures at 0.0 and 5.0 kbar. The ambient pressure experimental data is not available in the high pressure setup of the experiment. So we have used experimental structures at 1.18 and 6.15 kbar for plotting the displacement vectors. Key: C-Green, N-Red, and Ag-Blue.

To explore the possible origin of the above-mentioned behavior, the atomic displacement vectors (Figure 5) corresponding to the difference in the experimentally observed structure (Hodgson et al., 2014) at 1 and 6 kbar is compared with calculated structures at 0 and 5 kbar. It is found that experimentally the major difference comes from the displacement vector of Ag atoms, while computationally it is almost zero, i.e., no difference. There is also a significant difference in displacement patterns of the other atoms in the unit cell. Moreover, the experimentally observed atomic displacements are larger in magnitude compared to the calculated ones.

### Linear thermal expansion

**Figure 6:**
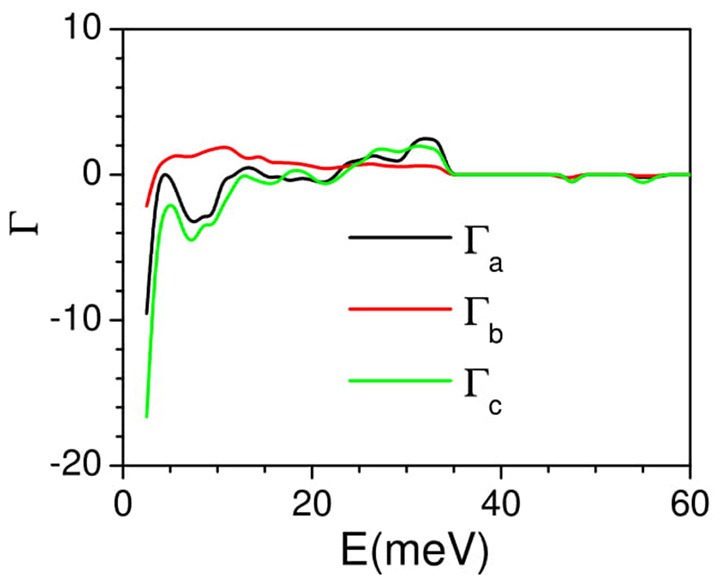
The calculated anisotropic mode Grüneisen parameters Γ_a_, Γ_b_, and Γ_c_, averaged over phonon modes in the entire Brillouin zone, as a function of phonon energy, on application of anisotropic pressure along a-, b-, and c-axes, respectively.

**Figure 7:**
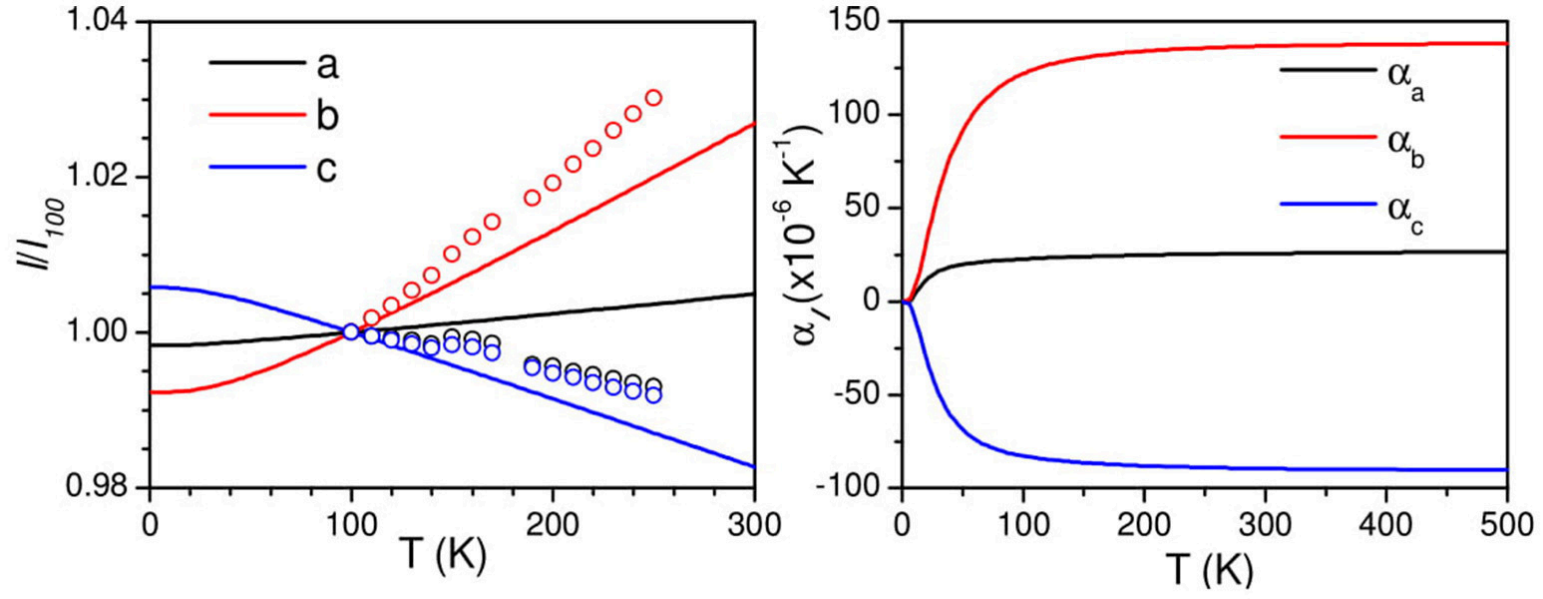
**(Left)** The calculated and experimental temperature dependence of unit cell parameters of AgC_4_N_3_. **(Right)** The calculated temperature dependence of linear thermal expansion coefficients of AgC_4_N_3_.

The linear thermal expansion behavior of AgC_4_N_3_ is calculated using the anisotropic pressure dependence of phonon spectra over the entire Brillouin zone within the framework of the quasiharmonic approximation. This methodology requires the complete elastic compliance tensor (section Anisotropic Compressibility Behavior) which is the inverse of the elastic constant tensor. The calculated anisotropic Grüneisen parameters are shown in Figure 6. The calculated low-energy mode Grüneisen parameters below 10 meV show negative values for the anisotropic stress along the *a* and *c-*axes, and possibly contributing to the negative thermal expansion in the ac plane. These also serve to explore thermal expansion behavior as discussed above. The calculated temperature dependence of the lattice parameters is compared with the experimental data. The calculated thermal expansion behavior (Figure 7) along the *b*- and *c*-axes agrees reasonably with the observation. The *c*-axis shows a negative linear thermal expansion while the b-axis shows a large positive expansion over the whole studied temperature range. However, the calculated *a*-lattice parameter shows a positive thermal expansion in contrast with the experiment. The calculated linear thermal expansion coefficients saturate above 200 K, with the values of α_a_ = 20 × 10^−6^ K^−1^, α_b_ = 125 × 10^−6^ K${}_{,}^{-1}$ α_c_ = −75 × 10^−6^ K^−1^. The calculated bulk volume thermal expansion agrees well with the experimental data.

**Figure 8:**
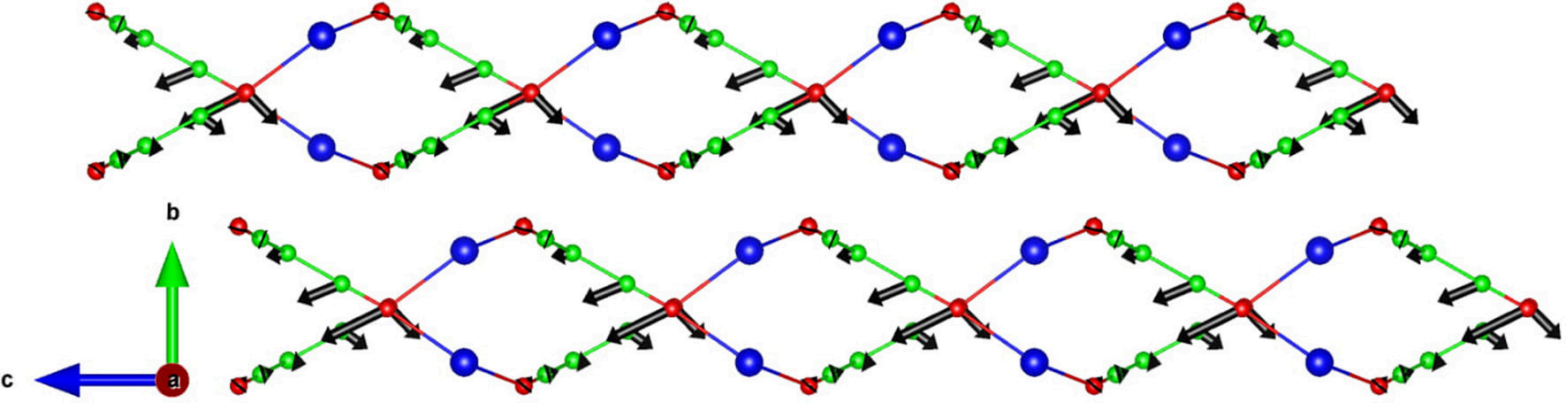
The calculated eigenvectors of the zone center phonon at 13.7 meV, giving rise to a negative thermal expansion behavior along the *c*-axis in AgC_4_N_3_. The corresponding mode Grüneisen values are Γ_a_ = 2.39, Γ_b_ = 0.10, Γ_c_ = −2.99. Key: C-Green, N-Red, and Ag-Blue.

The eigenvector pattern of specific phonon modes giving rise to the large negative thermal expansion along the *c*-axis is analyzed. The zone center mode with an energy of 13.7 meV (Figure 8) has Γ_a_ = 2.39, Γ_b_ = 0.10, Γ_c_ = −2.99. Assuming it corresponds to an Einstein mode with one degree of freedom, the contribution from this mode to the thermal expansion coefficients α_a_ = 11.34 × 10^−6^ K^−1^, α_b_ = 2.50 × 10^−6^ K^−1^, α_c_ = −11.89 × 10^−6^ K^−1^. As shown in Figure 8, only the atoms connected to C–N bonds have a larger amplitude and give rise to ripples in the layers along the *c*-axis, and is responsible for NTE along the *c*-axis.

In many metal organic frameworks, the experimentally observed anisotropic behavior in compressibility along a given crystallographic axis is found to lead to a fairly similar behavior in linear thermal expansion. The possible limitation of DFT to fully reproduce the compressibility behavior along the *a*-axis could be responsible for the difference in the thermal expansion behavior between the experiment and calculation. However, as far as the calculated phonon energies are concerned, these are correctly predicted by our *ab-initio* calculations at ambient pressure conditions.

## Conclusions

We have performed temperature dependent inelastic neutron scattering (INS) measurements to collect the temperature evolution (softening/hardening) of phonon spectra in the metal organic framework compound AgC_4_N_3_. *Ab-initio* lattice dynamical calculations are used to underpin the INS phonon data, over the entire Brillouin zone. The calculated partial density of states of various atoms is used to analyse the experimental spectra. The calculated anisotropic pressure dependent phonon spectra along with the estimated elastic constants are used to derive linear thermal expansion quantities of the compound. *Ab-initio* calculations performed at various uniform hydrostatic pressures are used to obtain the compressibility of the compound. Due to the structural peculiarity of AgC_4_N_3_, the *ab-initio* calculations including the van der Waals interaction could not reproduce the experimentally observed temperature/pressure dependence of “a” lattice parameter. However, the behavior of “b” and “*c*” lattice parameters is well-reproduced and calculations provide the microscopic mechanism of NTE and NLC along *c*-lattice parameter which arises from the rippling of structural sheets perpendicular to b-axis. It is clear that there is need for improvements in the van der Waals functions to accurately account for the structure and dynamics of metal organic framework compounds. Moreover, the experimentally measured phonon density of states will be very helpful for verification of any new van der Waals potentials in the coming future.

## Author contributions

RM formulated the problem, contributed in inelastic neutron scattering experiment, interpretation of experiment and *ab-initio* calculation, and writing of the manuscript. SC formulated the problem, contributed in interpretation of experiment and *ab-initio* calculation, and writing of the manuscript. BS and MG contributed in inelastic neutron scattering experiment, *ab-initio* calculations, interpretation of experiment and *ab-initio* calculation, and writing of the manuscript. MZ and HS participated in inelastic neutron scattering experiment and data analysis. SH and AG synthesized and characterized the AgC_4_N_3_ samples.

### Conflict of interest statement

The authors declare that the research was conducted in the absence of any commercial or financial relationships that could be construed as a potential conflict of interest.
